# Factors associated with prolonged COVID-related PTSD-like symptoms among adults diagnosed with mild COVID-19 in Poland

**DOI:** 10.3389/fpsyg.2024.1358979

**Published:** 2024-03-14

**Authors:** Sapir Elkayam, Emilia Łojek, Marcin Sękowski, Dominika Żarnecka, Anna Egbert, Julia Wyszomirska, Karolina Hansen, Ewa Malinowska, Lucette Cysique, Bernice Marcopulos, Natalia Gawron, Marta Sobańska, Małgorzata Gambin, Paweł Holas, Agnieszka Pluta, Sylwia Hyniewska

**Affiliations:** ^1^Faculty of Psychology, University of Warsaw, Warsaw, Poland; ^2^Department of Psychology, The Maria Grzegorzewska University, Warsaw, Poland; ^3^Psychology Department, St. Joseph's University New York, Brooklyn, NY, United States; ^4^Department of Psychology, Medical University of Silesia, Katowice, Poland; ^5^School of Psychology, Faculty of Science, The University of New South Wales, Sydney, NSW, Australia; ^6^Department of Graduate Psychology, James Madison University, Harrisonburg, VA, United States; ^7^School of Medicine, Department of Psychiatry and Neurobehavioral Sciences, University of Virginia, Charlottesville, VA, United States

**Keywords:** COVID-19, posttraumatic stress, neurocognitive symptoms, emotional functioning, stigma, social support

## Abstract

COVID-19 has been considered a possible cause of post-traumatic stress disorder (PTSD) or similar conditions. However, what specific disease symptoms may contribute most to prolonged PTSD-like symptoms in COVID-19 survivors is unclear. The study aimed to present the factor structure of COVID-19 symptoms and identify which symptoms of COVID-19 best explain the subsequent presence of PTSD-like symptoms in mild COVID-19 survivors. COVID-positive adults (*n* = 341) completed online self-report scales at the baseline assessment (T1) and after approximately 4 months (T2), including The Patient Health Questionnaire Anxiety-Depression Scale; The Scale of Psychosocial Experience Related to COVID-19, The Primary Care PTSD Screen for DSM-5; and self-designed questionnaires evaluating the severity of COVID-related medical and neurocognitive symptoms and pre-pandemic variables. Exploratory factor analysis revealed five factors of COVID-19 symptoms: flu-like, respiratory, cold, neurological, and neurocognitive. Hierarchical logistic regression showed that besides selected control variables (anxiety and depression, presence of PTSD-like symptoms, COVID-related stigma in T1), neurocognitive symptoms of COVID-19 in T1 but not other symptoms of the disease were a significant predictor of the presence of PTSD-like symptom in T2. Findings suggest a need for a comprehensive neurocognitive assessment of people diagnosed with COVID-19 and prompt interventions targeting the prevention of potential risks for long-term PTSD-like states at the community level.

## Introduction

The COVID-19 infectious disease was designated a global pandemic by the World Health Organization (WHO) on March 11, 2020 (Adil et al., 2021). Since its emergence, millions of confirmed cases and fatalities have been recorded worldwide (World Health Organization, 2023). SARS-CoV2 infection can severely affect one’s health, disrupting body functioning on different levels and leading to severe psychological distress and disability, especially among vulnerable groups (Zhu et al., 2023).

The most common COVID-19 symptoms are divided into three main categories: cardiorespiratory (fatigue, chest pain, dyspnea, sore muscle, headaches, and palpitations), inflammatory (e.g., muscle pain, weakness, sleep disorders, hair loss, dizziness, and gastrointestinal problems) and neurological symptoms (e.g., anosmia, headaches, neuropathies, dizziness, paresthesia, and problems with vision and with balance; Kuodi et al., 2023). However, studies based on factor analysis suggest a slightly greater complexity of the symptoms of the disease. For example, Pinto et al. (2022) showed five factors of COVID-19 symptoms: cold and flu-like symptoms, change in smell and/or taste, dyspnea and chest pain, cognitive and visual problems, and cardiac symptoms. These results suggest an important group of disease symptoms—broad neurocognitive symptoms.

Studies have proven the existence of so-called *long-COVID* or *post-COVID syndrome*, referring to persistent difficulties experienced by patients long after recovery (Lu et al., 2020). These difficulties are not only strictly medical but also emotional and neurocognitive. COVID-19 survivors report experiencing brain fog, loss of concentration, altered smell, sleep disturbances, and fatigue (Kuodi et al., 2023). Subjective neurocognitive difficulties are present in around 70% of the people recovering from COVID-19 (De Luca et al., 2022), with the most described affected cognitive domains including perception of taste and smell, attention, memory, learning, and executive functions (Almeria et al., 2020). Neurocognitive difficulties can be a novel, unexpected challenge to everyday functioning that worsen quality of life and increase psychological distress (Cysique et al., 2021; Maggi et al., 2022).

Post-traumatic stress disorder (PTSD) has also been considered one of the potential long-term consequences of COVID-19 (Schou et al., 2021; Bajoulvand et al., 2022). Exposure to infectious diseases such as COVID-19 poses a unique, multidimensional severe stressor that can be classified into several sources: direct (e.g., life-threatening experience of physical discomfort) and indirect (e.g., exposure witnessing others struggle against the disease; being subjected to exclusion and stigmatization; Xiao et al., 2020). Although the status of some of these experiences as traumatic stressors is controversial because they are not events that threaten the life or integrity of the person (Sekowski et al., 2021), all of these experiences can result in the development of prolonged symptoms similar or identical to PTSD symptoms such as persistent intrusion, increased alertness, avoidance behavior, and adverse effects on mood and cognition associated with the traumatic stimuli (Ochnik et al., 2022).

Risk factors for PTSD (or PTSD-like conditions) during the pandemic are, among others, younger age, female gender, history of mental health problems (Liang et al., 2020; Yunitri et al., 2022), and receiving intensive care for severe COVID-19 illness (Rogers et al., 2020). Additionally, stigma experiences, even among people with mild COVID-19, have previously been reported and linked to PTSD and PTSD-like symptoms (Kang et al., 2021). In contrast, social connectedness and support are prominent protective factors against mental health difficulties, including PTSD, due to their central role in facilitating people’s adaptive coping abilities and recovery from adversities (Matos et al., 2021).

PTSD symptoms often co-occur with neurocognitive difficulties, such as difficulties with psychomotor and information processing speed, attention span, learning, memory, and word-finding (Kertzman et al., 2014; Vasterling et al., 2019). Studies have found significant structural changes in the brains of PTSD patients affecting, e.g., the prefrontal cortex, hippocampus, amygdala, and anterior cingulate cortex (Vasterling et al., 2019; Benedetti et al., 2021), which are associated with a variety of cognitive functions. Importantly, impaired cognitive functions may constitute a significant risk factor for PTSD (Schultebraucks et al., 2022). Research on PTSD and cognitive issues post-COVID-19 must consider this complex relationship between trauma and cognitive complaints. A similar relationship has been found between mild traumatic brain injury and PTSD in combat veterans (Mattson et al., 2019).

Given the limited understanding of how distinct groups of COVID-19 symptoms relate to the development of prolonged PTSD-like symptoms, especially in mild cases of infection, this study set out to answer two questions. First, what is the structure of COVID-19 symptoms in a sample of adults with mild COVID-19 infection? Second, which groups of COVID-19 symptoms predict the presence of prolonged COVID-related PTSD-like symptoms, in addition to and beyond selected sociodemographic, psychosocial (depression and anxiety, COVID-related stigma, and social support), and clinical (pre-existing conditions) factors? Specifically, we were interested in the role of COVID-related neurocognitive symptoms in explaining prolonged PTSD-like symptoms. We hypothesized that COVID-19 neurocognitive symptoms will explain COVID-related PTSD-like symptoms besides other potential predictors of PTSD-like symptoms included in the study.

## Materials and methods

### Procedure and recruitment

The study was approved by the Institutional Review Board of the Faculty of Psychology, University of Warsaw. The findings are part of a larger research project on neuropsychological functioning and quality of life of people after COVID-19 that was conducted between May 2020 and June 2022 (Łojek et al., 2021; Malinowska et al., 2022). The study consisted of a baseline (T1) and a follow-up (T2) assessment at approximately 4-month interval.

Participants were recruited through social media, informal social networks, and snowball sampling. Through this method, 1,038 responses were collected. However, only 96 participants with complete data from two assessments were selected from this sample for the current study. These data were collected in the early period of the pandemic (May 2020–October 2021). In addition, in the later period (February–June 2022), we cooperated with the Nationwide Research Panel Ariadna (Ogólnopolski Panel Badawczy Ariadna), which hosts an opt-in online panel of active panelists with verified profiles to enlarge the number of participants. Through this method, 683 responses were collected, of which 245 participants with complete data from both assessments were included in the study. Participants reviewed an information letter and completed a consent form. Inclusion criteria for this study were: aged ≥18 years, based in Poland at the time of the assessment, and reporting having at least one laboratory antigen or serological test for COVID-19 confirming the infection.

### Participants

A total of 341 participants completed two assessments forming the sample of this study (aged 18–86; *M* = 42.89, *SD* = 13.82; 59.8% female).

The participants entered the project in two different periods of the pandemic, characterized by various socio-political and epidemiological contexts, which could impact their mental health. Therefore, we compared results from T1 between the samples recruited in the first and second data collection periods. There were no differences between the samples measured in the first (*M* = 13.85, *SD* = 10.62) and second (*M* = 12.43, *SD* = 11.18) periods of the pandemic in emotional functioning [*t*(339) = 1.074, *p* = 0.284]. There also was no difference between the samples measured in the first (*M* = 6.01, *SD* = 2.79) and second (*M* = 5.85, *SD* = 2.97) periods of the pandemic in stigma [*t*(339) = 0.495, *p* = 0.65]. The only significant difference was found between the samples in PTSD-like symptoms [*χ^2^*(1,341) = 7.25, *p* = 0.007], indicating that more participants from the early time of the pandemic (53%) reported at least one symptom than no symptoms compared to the sample evaluated in the later time of the pandemic (37%). The above results indicate that despite differences in PTSD-like symptoms, the overall emotional and social functioning at baseline of both samples were comparable. Therefore, their data were not separated in further analyses.

Detailed sociodemographic and pre-existing clinical characteristics of the participants are displayed in Table 1.

**Table 1:** Sample sociodemographic and pre-existing clinical characteristics.

Variable	*n*	%
Age (*M* ± *SD*)	42.89 ± 13.82	
Gender		
Male	137	40.2
Female	204	59.8
Education		
Primary	64	18.8
Secondary	97	28.4
Higher	180	52.8
Employment		
Employed	272	79.8
Unemployed	15	4.4
Student	14	4.1
Retired, receiving disability aid/benefit or a homemaker	40	11.7
Residence		
Rural	64	18.8
Semi-rural	97	28.4
Urban	180	52.8
Pre-existing medical condition^a^		
None	296	86.8
Endocrine disorder	20	5.9
Hypertension	15	4.4
Autoimmune condition	14	4.1
Lung disease	5	1.5
Ischemic heart disease	5	1.5
Circulatory failure	3	0.9
Cancer	2	0.6
Epilepsy	1	0.3
Stroke	1	0.3
Brain concussion	1	0.3
Dementia	1	0.3
Other	13	3.8
Pre-existing mental health condition^a^		
None	330	96.8
Mood disorder	8	2.3
Anxiety disorder	6	1.8

^a^Percentages may sum to more than 100% as respondents could select more than one condition.

### Measures

An online questionnaire was designed for the current study and consisted of several parts, including:

#### Demographics

This part of the questionnaire included questions on participants’ age, gender, education level, employment status, community type, financial situation, and household size.

#### Medical background

Medical information was gathered, including earlier diagnoses of conditions influencing the brain, such as neurological, oncological, endocrine, pulmonary, and cardiological diseases. This part also included questions about pre-pandemic anxiety or mood disorders and smoking, alcohol consumption, and substance use patterns.

#### Severity of COVID-related symptoms

The researchers generated a list of 19 COVID-19 symptoms based on the WHO COVID-19 case definition (World Health Organization, 2022). The list included the following symptoms: sore throat, cough, runny nose, diarrhea, vomiting, conjunctivitis, chest pain, shortness of breath, fever, weakness, dizziness, headaches, fatigue, problems with balance and gait, stroke, epileptic seizures, vision impairment, nerve pain, and skeletal muscle pain. Additionally, participants completed 22 questions about neurocognitive symptoms they possibly experienced in connection to COVID-19. The items were designed based on the Patient’s Assessment of Own Functioning (PAOFI; Chelune et al., 1986) and the Smell and Taste scale of the National Health and Nutrition Examination Survey 2013–2014 (NHANES; Centers for Disease Control and Prevention, 2013). The questions covered six cognitive domains: (i) smell and taste (e.g., detecting smells, odor recognition, detecting flavors, and flavors recognition); (ii) use of hands (e.g., carrying out activities using the right or left hand, including writing, getting dressed, and lifting and moving objects); (iii) information processing (e.g., slower information processing); (iv) high level cognitive and intellectual functions (e.g., finishing tasks, planning and organizing, following instructions, solving problems, and confusing or illogical thoughts); (v) memory (e.g., recalling names and events); and (vi) language and communication (e.g., understanding speech and written text). Participants were asked to indicate whether they experienced difficulties due to the virus for each item. If they experienced the symptom, participants were asked to rate on a five-point scale ranging from 1 = “mild” to 5 = “severe” how difficult the symptom was to them.

#### Depression and anxiety

To measure emotional functioning, the Patient Health Questionnaire Anxiety-Depression Scale (PHQ-ADS; Kroenke et al., 2016) was applied. PHQ-ADS is a self-report questionnaire consisting of the Patient Health Questionnaire (PHQ-9) and the Generalized Anxiety Disorder Scale (GAD-7), providing a composite score of depression and anxiety. The PHQ-ADS consists of 16 items, which assess the presence and severity of both anxiety and depressive symptoms over the past 2 weeks. The first seven items measure anxiety symptoms (e.g., feeling nervous or anxious, worrying too much about different things), and the next nine items measure depression symptoms (e.g., feeling down, depressed, or hopeless). Each item is rated on a four-point scale (0 = “not at all” to 3 = “nearly every day”), with higher scores indicating greater symptom severity over the past 2 weeks. The PHQ-ADS has been found to have good reliability and validity in various populations, including Polish adults (Gambin et al., 2021). Cronbach’s alpha in the current study was 0.95.

#### Social support and stigma

A scale of nine questions was designed to measure psychosocial experiences related to COVID-19 (The Scale of Psychosocial Experiences Related to COVID-19; SPER-COVID-19), including positive (receiving social support) and negative (stigma related to disease) subjective experiences. The respondent answers each question on a scale from 1 = “never” to 5 = “always.” Five items measure the support received, including the ability to share concerns with loved ones, belief in the readiness of others to provide financial support if needed, feeling loved and wanted, receiving information and advice on dealing with the pandemic, and the presence of others who can help with the respondent’s daily affairs. The other four items relate to experienced stigma, such as feeling that others blame the respondent for being infected, that others are unkind to the respondent that others have avoided the respondent since the illness, and feeling embarrassed about being infected. The scale was suitable for factor analysis [KMO = 0.829; Bartlett test (*χ2* = 1450.04, *df* = 36, *p* < 0.001)], and Exploratory Factor Analysis (EFA) with oblimin rotation showed two factors explaining 67.35% of the variance and covering items measuring social support (five items with factor loadings between 0.831 and 0.703; *α* = 0.84) and stigma (four items with loadings between 0.916 and 0.779; *α* = 0.88), respectively.

#### Post-traumatic stress disorder

To measure PTSD symptoms, we used The Primary Care PTSD Screen for DSM-5 (PC-PTSD-5; Prins et al., 2016). PC-PTSD-5 is a short screening tool consisting of five questions that aim to identify possible cases of PTSD in primary care settings. The first question evaluates whether the individual has experienced a traumatic event in their lifetime. If they respond positively, they are then asked five additional questions about how the trauma has impacted them in the last month. The answers are scored on a scale of 0 (no symptom) to 1 (presence of the symptom). Items refer to PTSD symptoms such as nightmares and unwanted thoughts about a traumatic event, avoiding reminders, excessive vigilance, numbness/detachment, and blaming yourself. In the current survey, respondents were asked whether these symptoms occurred in relation to their COVID-19 disease and/or pandemic. Since we modified the tool to validate its internal validity in our study, we performed an EFA with oblimin rotation. The results of Bartlett’s sphericity test (*χ^2^* = 352.43, *df* = 10, *p* < 0.001) and the KMO test (0.796) showed that the results in the PC-PTSD-5 are suitable for factor analysis. All five items formed one factor with eigenvalues >1 (factor loadings from 0.78 to 0.67; *α* = 0.75), accounting for 50.47% of the variance. Since we obtained a strongly left-skewed distribution of scores with zero inflation, the scale scores were dummy-coded (1 = presence of at least one PTSD symptom; 0 = no symptom).

### Data analysis

Analyses were conducted using IBM SPSS Statistics version 28. First, EFA with oblimin rotation was performed to extract COVID-related symptom groups. Next, a hierarchical logistic regression model was constructed to reveal the impact of psychosocial and clinical factors (predictors introduced in the first step of the analysis) and the severity of COVID-related medical (added in the second step) and neurocognitive symptoms (the third step) in T1 on the presence of PTSD-like symptoms in T2. The model’s likelihood-ratio chi-square (χ^2^) test (in subsequent analysis steps) versus the model from the previous regression step was used to determine whether the current model outperforms the previous model in explaining the variance of the outcome variable.

## Results

### Factor structure of symptoms of mild COVID-19

Table 2 summarizes the EFA results for COVID-19 symptoms. One symptom with zero occurrence (stroke) was excluded from the analysis. The KMO value was 0.88, and Bartlett’s test of sphericity was significant (*χ2* = 4211.956, *df* = 561, *p* < 0.001), indicating that the data were suitable for factor analysis. The results suggested a nine-factor model with eigenvalues >1, explaining 60.32% of the total variance. Factor 6 included epileptic seizures, vision impairment, and diarrhea. However, we excluded it from further analysis due to unacceptable reliability (*α* = 0.24). Factors 7, 8, and 9 each included a single item (vomiting, problems with balance or gait, and conjunctivitis) and were thus excluded from further analyses.

**Table 2:** Results of exploratory factor analysis (EFA).

COVID-related symptoms	Flu-like symptoms	Respiratory symptoms	Cold symptoms	Neurological symptoms	Neurocognitive symptoms
Medical					
Weakness	0.788				
Fatigue	0.672				
Skeletal muscle pain	0.605				
Fever	0.521				
Chest pain		0.815			
Shortness of breath		0.782			
Sore throat			0.778		
Runny nose			0.742		
Cough			0.508		
Nerve pain				0.700	
Headache				0.584	
Dizziness				0.558	
Neurocognitive					
Remembering events					0.783
Solving problems					0.773
Information processing					0.771
Distraction					0.745
Confusing or illogical thoughts					0.737
Following instructions					0.729
Understanding speech					0.727
Planning and organizing					0.718
Recalling names					0.715
Finishing tasks					0.706
Motivation					0.697
Performing activities					0.689
Understanding written text					0.665
Irritation					0.607
Use of hands					0.447
Smell and taste					0.419
% of variance	7.54	4.8	4.35	3.29	25.99

Only factor loadings > 0.40 are presented.

The final five-factor structure is presented in Table 2. Based on the content analysis of items forming factors 1–5, they were named as follows: Neurocognitive symptoms (*α* = 0.75); Flu-like symptoms (*α* = 0.75); Respiratory symptoms (*α* = 0.68); and Cold symptoms (*α* = 0.67). Neurological symptoms had lower, yet still acceptable, reliability (*α* = 0.51; Pallant, 2010). Based on the data shown in Supplementary Table S1, the poor internal consistency of the Neurological factor may be because, although it is created by symptoms of nervous system disorders (nerve pain, headaches, and dizziness), participants might experience single symptoms but not a whole set of symptoms of this category. The small number of items may have also contributed to the lower reliability. Despite these limitations, the factor was included in further analysis owing to the theoretical relevance and consistency with past research on COVID-related symptoms (e.g., Luo et al., 2020; Dixon et al., 2021; Williams and Zis, 2023).

### T1 predictors of PTSD-like symptoms in T2

**Figure 1:**
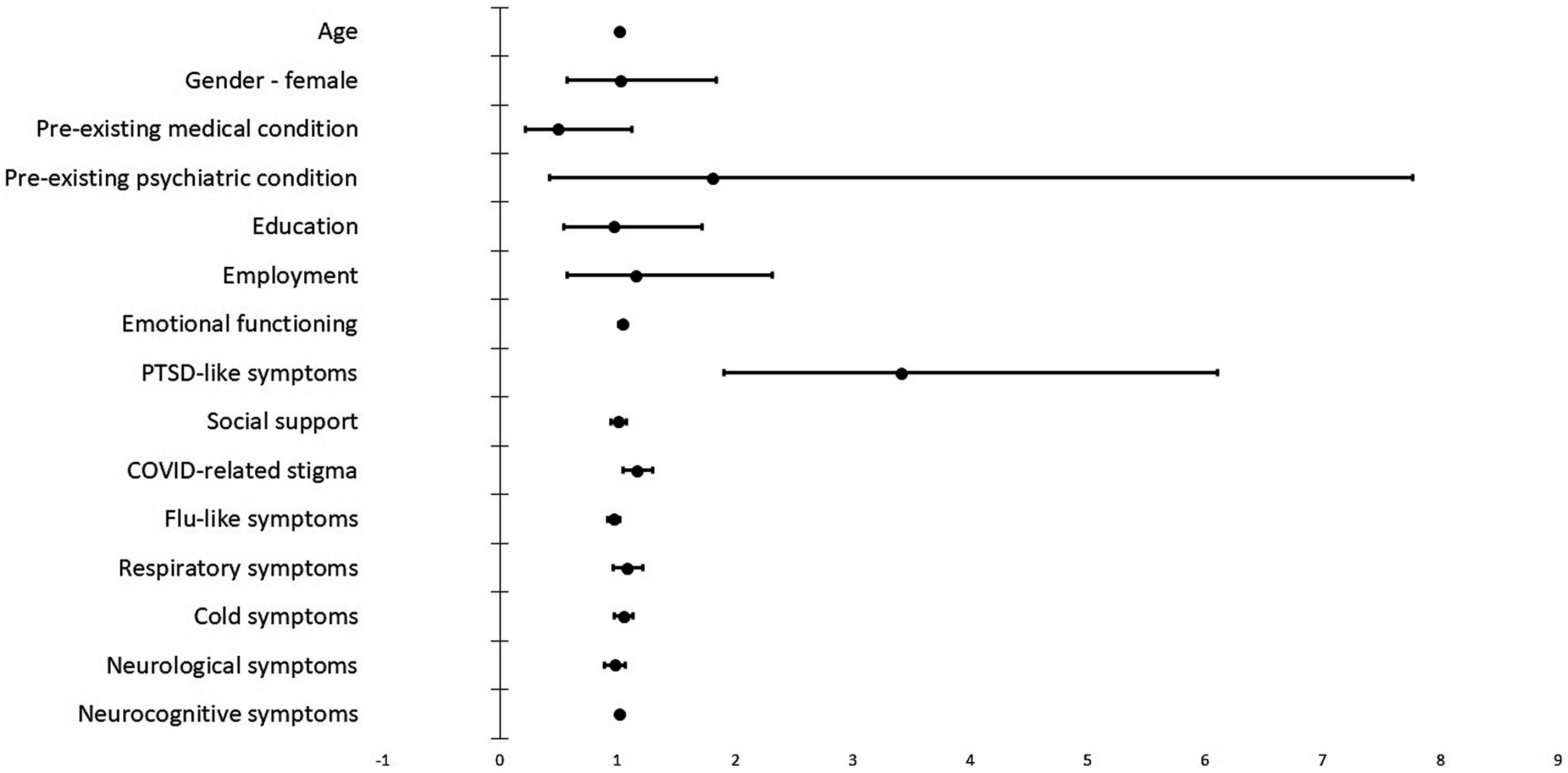
Regression coefficients for the T1 variables predicting PTSD-like symptoms in T2. The image shows the exponentiation of the B coefficient (odds ratio) from the logistic regression and the 95% confidence intervals.

Of the respondents, 142 (42%) and 138 (41%) had at least one PTSD-like symptom at T1 and T2, respectively. Table 3 and Figure 1 display the regression coefficients, odds ratios, 95% confidence intervals for odds ratios, and Nagelkerke *R^2^* for the final step of the model. Including demographic and clinical variables did not significantly improve the null model, χ^2^(6) = 4.54, *p* = 0.60. Adding psychosocial variables improved the model, χ^2^(4) = 95.36, *p* < 0.001, with poorer emotional functioning (stronger depressive and anxiety symptoms), presence of PTSD-like symptoms, and a higher level of COVID-related stigma in T1 predicting the presence of PTSD-like symptoms in T2. Adjusting for the severity of subgroups of COVID-related medical symptoms did not improve the model, χ^2^(4) = 3.75, *p* = 0.44. Finally, the severity of COVID-related neurocognitive symptoms in T1 improved the model, χ^2^(1) = 3.96, *p* = 0.047, significantly predicting the presence of PTSD-like symptoms in T2.

**Table 3:** Descriptive statistic and final model of hierarchical logistic regression analysis predicting PTSD-like symptoms in T2.

			PTSD-like symptoms (T2)			
Predictors (T1)	*M*	*SD*	*Exp(B)*	*SE*	*OR*	95% CI
Demographic and clinical						
Age	42.89	13.82	1.01	0.011	1.010	[0.98, 1.03]
Gender^a^			1.026	0.298	1.026	[0.57, 1.84]
Education^b^			0.969	0.293	0.969	[0.54, 1.72]
Employment^c^			1.15	0.358	1.150	[0.57, 2.31]
Pre-existing medical condition^d^			0.488	0.425	0.488	[0.21, 1.12]
Pre-existing psychiatric condition^d^			1.812	0.743	1.810	[0.42, 7.76]
Psychosocial						
Emotional functioning^e^	12.89	11.02	1.04^**^	0.015	1.040	[1.01, 1.07]
PTSD-like symptoms^f^			3.413^***^	0.296	3.413	[1.90, 6.10]
COVID-related stigma	5.89	2.91	1.164^**^	0.054	1.164	[1.04, 1.29]
Social support	18.50	4.33	1.005	0.032	1.005	[0.94, 1.07]
Severity of COVID-related medical symptoms						
Flu-like symptoms	9.15	5.89	0.968	0.027	0.968	[0.91, 1.02]
Respiratory symptoms	1.30	2.38	1.078	0.059	1.078	[0.95, 1.21]
Cold symptoms	3.58	3.56	1.05	0.039	1.050	[0.97, 1.13]
Neurological symptoms	3.03	3.39	0.976	0.047	0.976	[0.89, 1.07]
Severity of COVID-related neurocognitive symptoms	20.67	20.18	1.016*	0.008	1.016	[1.00, 1.03]
Nagelkerke *R*^2^			0.365			

OR, Odds ratio; CI, Confidence interval.

^a^0 = male, 1 = female.

^b^0 = primary or secondary education, 1 = higher education.

^c^0 = unemployed, student, homemaker, retired or receives disability/aid benefit, 1 = employed.

^d^0 = no condition, 1 = at least one condition.

^e^A composite score of depression and anxiety.

^f^0 = no symptoms, 1 = at least one symptom.

^*^p < 0.05; ^**^p < 0.005; ^***^p < 0.001.

Summarizing, as one can also see from Table 3 and Figure 1, significant predictors of PTSD-like symptoms after COVID-19 were depression and anxiety symptoms (odds ratio around 1, but with small confidence intervals), PTSD-like symptoms at T1 (large variability/CI, but a high odds ratio), and COVID-related stigma and the mean of the neurocognitive symptoms.

## Discussion

The study set out with the aims of investigating the structure of COVID-19 symptoms among adults with mild COVID-19 infection and identifying groups of COVID-19 symptoms that can be potential risk factors associated with prolonged COVID-related PTSD-like symptoms in addition to sociodemographic and clinical variables. To achieve our goals, we conducted a study on a Polish sample of COVID-19 survivors who developed mild disease symptoms. Perhaps the most striking finding is that experiencing neurocognitive symptoms such as difficulties with memory and thinking after mild COVID-19 infection was strongly linked to the presence of prolonged PTSD-like symptoms. Additionally, anxiety, depression, and COVID-related stigma also predicted the likelihood of experiencing these symptoms in the subsequent months.

Exploratory Factor Analysis showed that COVID-19 symptoms in our sample form five groups of symptoms: flu-like, respiratory, cold, neurological, and neurocognitive symptoms. Earlier studies on factor analysis revealed a similar structure of COVID-19 symptoms (Luo et al., 2020; Pinto et al., 2022). However, our study considered more potential neurocognitive symptoms of COVID-19 than previous studies. By showing that as many as 16 neurocognitive symptoms form a separate factor of COVID-19 symptoms, we additionally supported the validity of separating this subgroup of disease symptoms.

Our study confirmed the positive association between the severity of COVID-related neurocognitive symptoms at T1 and the presence of PTSD-like symptoms in a 4-month follow-up. However, it did not demonstrate the predictive role of the severity of COVID-related medical symptoms (flu-like, respiratory, cold, and neurological). Moreover, the model including COVID-19 neurocognitive symptoms as an additional predictor significantly better explained the presence of PTSD-like symptoms scores than the model considering only sociodemographic and clinical, psychosocial factors and other COVID-19 symptoms. These results match those observed in previous studies. For example, Maggi et al. (2022) found that subjective cognitive complaints at baseline were one of the most influential factors associated with PTSD after 2 months.

One may speculate that the experience of COVID-related neurocognitive deficits, particularly in individuals with mild symptoms of the disease, was unexpected and highly stressful compared to other symptoms and may intensify their negative impact, making them a robust stressor contributing to PTSD or PTSD-like symptoms above other well-documented symptoms of COVID-19. In addition, the impact of neurocognitive deficits on daily functioning, such as performance at work, managing everyday tasks, and maintaining social connections, could make them more disruptive than other symptoms. Finally, PTSD and neuropsychological symptoms are associated with similar brain regions, and there may be overlapping neurofunctional mechanisms involved in their development (Vasterling et al., 2019).

Our results can be interpreted in light of Lazarus and Folkman’s (1984) transactional stress and coping theory. Limited medical, social, and individual resources to cope with an unknown, life-threatening disease, as well as social isolation and stigma, caused considerable stress in infected individuals, even with mild symptoms. Moreover, a decline in cognitive abilities (or the subjective belief in a decline) could make it difficult to access appropriate coping methods. This could exacerbate their stress and psychological trauma.

The final regression analysis model demonstrated that elevated levels of anxiety and depression, the existence of PTSD-like symptoms, and the presence of COVID-related stigma at T1 are indicative of the likelihood of experiencing PTSD-like symptoms in the subsequent 4-month follow-up assessment. Previous research also showed that COVID-19 exacerbates psychological conditions such as anxiety and depression (e.g., Dacosta-Aguayo et al., 2022), and those, in turn, increase the likelihood of developing PTSD and similar conditions. Furthermore, as a consequence of labeling and harmful stereotyping due to the novelty, uncertainty, and person-to-person transmission of the virus (i.e., stigma), one may experience mental health problems, including extreme stress (Kang et al., 2021).

The findings of the study must be considered within its limitations. Firstly, our study was conducted in the context of changing epidemiological and socio-political situations (i.e., the war in Ukraine), and we did not include variables controlling these factors. Secondly, the study included a small and specific cohort of COVID-19 survivors based in Poland, which limits its generalizability. Additionally, the use of self-reported measures may result in certain biases, such as recall errors or social desirability, which could potentially compromise the validity and reliability of the data. Finally, although attempts were made to adjust assessment tools for the study group, such as modifying the PC-PTSD-5, the validity of these instruments in the context of COVID-19 may still be questionable.

## Conclusion

The study highlights the multifactorial determinants contributing to prolonged PTSD-like symptoms following mild COVID-19. These factors include emotional symptoms (anxiety, depression, and PTSD) during the illness course, social stigma due to the infection and subjective perceptions of neurocognitive deficits (e.g., smell and taste, information processing, memory, thinking, and verbal communication). It further illustrates that among the symptoms of mild COVID-19, only the reported impairment in neurocognitive functions emerged as significant for the persistence of PTSD-like symptoms. This was observed among individuals who were healthy before COVID-19, that is, had no prior diagnosis of any serious mental or physical conditions. According to Greißel et al. (2024), these participants may have had a predisposition for developing PTSD-like symptoms post-COVID-19.

The close association observed between the prevalence of PTSD or PTSD-like symptoms and neurocognitive deficits, as well as with shared neurobiological mechanisms of both conditions, suggests that prolonged PTSD symptoms and similar conditions after COVID-19 may potentially involve long-term neurocognitive deficits. Therefore, an essential component of the treatment and therapy of individuals with prolonged PTSD-like symptoms after COVID-19 should include a comprehensive neuropsychological assessment and, where necessary, neuropsychological rehabilitation. Neuropsychological intervention could thus serve as a crucial element in effectively treating and preventing mental health disorders in people after COVID-19. Unfortunately, in many countries, including Poland, neuropsychological services are insufficiently accessible or under-recognized by healthcare professionals. There is a clear need to improve this situation in healthcare systems for the benefit of the potentially large number of people suffering from undiagnosed mental disorders after COVID-19 that may negatively impact functioning in everyday life. This can be done by including neuropsychological examination as a standard procedure in medical services offered to individuals after COVID-19 by healthcare systems worldwide.

## Data availability statement

The raw data supporting the conclusions of this article will be made available by the authors, without undue reservation.

## Ethics statement

The study was approved by the Institutional Review Board of the Faculty of Psychology, University of Warsaw. Participants reviewed an information letter and completed a consent form.

## Author contributions

SE: Conceptualization, Data curation, Formal analysis, Visualization, Writing – original draft, Writing – review & editing. EŁ: Conceptualization, Formal analysis, Funding acquisition, Methodology, Project administration, Supervision, Writing – review & editing. MSę: Conceptualization, Formal analysis, Methodology, Writing – original draft, Writing – review & editing, Validation. DŻ: Data curation, Investigation, Writing – original draft. AE: Data curation, Investigation, Methodology, Project administration, Writing – review & editing. JW: Investigation, Resources, Writing – original draft. KH: Methodology, Project administration, Software, Visualization, Writing – review & editing. EM: Investigation, Methodology, Project administration, Resources, Writing – review & editing. LC: Conceptualization, Supervision, Writing – review & editing. BM: Conceptualization, Supervision, Writing – review & editing. NG: Investigation, Methodology, Writing – review & editing. MSo: Investigation, Methodology, Writing – review & editing. MG: Writing – review & editing, Conceptualization, Investigation, Methodology. PH: Conceptualization, Methodology, Writing – review & editing. AP: Investigation, Methodology, Writing – review & editing. SH: Methodology, Writing – review & editing, Conceptualization, Investigation.
