# Supplementary material for: Factors associated with prolonged COVID-related PTSD-like symptoms among adults diagnosed with mild COVID-19 in Poland
Source: Front Psychol. 2024 Mar 14;15:1358979. doi: 10.3389/fpsyg.2024.1358979 (PMC10973129; doi:10.3389/fpsyg.2024.1358979)
Supplement: Supplementary file 1 [file Table_1.pdf]

Supplementary Material

Table S1. *Descriptive statistics of COVID-related medical and neurocognitive symptoms subjectively evaluated by participants on a 5-point severity scale* [a]

Symptom	*M*	*SD*	Min-Max	Skewness	Kurtosis
Weakness	2.72	2.01	0-5	-0.381	-1.503
Fatigue	2.76	1.99	0-5	-0.413	-1.433
Skeletal muscles pain	1.77	2.04	0-5	0.462	-1.520
Fever	0.96	1.11	0-5	0.97	0.441
Chest pain	0.68	1.39	0-5	1.751	1.466
Shortness of breath	0.62	1.37	0-5	1.99	2.515
Sore throat	0.8	1.45	0-5	1.487	0.664
Runny nose	1.13	1.66	0-5	1.016	-0.565
Cough	1.65	1.82	0-5	0.494	-1.321
Nerve pain	0.41	1.22	0-5	1.221	5.949
Headaches	1.96	2.03	0-5	0.261	-1.658
Dizziness	0.66	1.4	0-5	1.899	2.097
Epileptic seizures	0.02	0.24	0-4	14.830	230.395
Vision impairment	0.15	0.73	0-5	5.046	24.905
Diarrhea	0.4	1.11	0-5	2.79	6.829
Vomiting	0.07	0.51	0-5	8.656	77.681
Problems with balance and gait	0.23	0.9	0-5	3.858	13.888
Conjunctivitis	0.13	0.67	0-5	5.658	33.340
Remembering events	1.03	1.49	0-5	1.019	-0.533
Solving problems	0.67	1.26	0-5	1.638	1.278
Information processing	1.07	1.49	0-5	1.057	-0.193
Distraction	0.78	1.39	0-5	1.426	0.616
Confusing or illogical thoughts	0.77	1.37	0-5	1.508	0.896
Following instructions	0.52	1.16	0-5	2.061	2.934
Understanding speech	0.6	1.24	0-5	1.916	2.333
Panning and organizing	0.74	1.35	0-5	1.633	1.394
Recalling names	0.98	1.5	0-5	1.187	-0.025
Finishing tasks	0.6	1.21	0-5	1.806	1.936
Motivation	1.17	1.63	0-5	0.943	-0.630
Performing activities	1.23	1.59	0-5	0.773	-0.978
Understanding written text	0.4	1.05	0-5	2.555	5.408
Irritation	0.92	1.47	0-5	1.274	0.184
Use of hands	0.3	0.8	0-4	2.812	7.256
Smell and Taste	1.43	1.61	0-5	0.757	-0.759

Note. n = 341; [a] Scoring range: 0 – no symptoms, 1 – mild, 2 – mild to moderate, 3 – moderate, 4 – moderate to severe, 5 – severe
